# Anxiety and Extraversion in Lupus-Related Atherosclerosis

**DOI:** 10.3389/fpsyt.2018.00246

**Published:** 2018-06-19

**Authors:** Maira Giannelou, Dimitrios Tseronis, Eleni Antypa, Clio P. Mavragani

**Affiliations:** ^1^Department of Pathophysiology, School of Medicine, National and Kapodistrian University of Athens, Athens, Greece; ^2^Department of Rheumatology, General Hospital of Athens “G. Gennimatas”, Athens, Greece; ^3^Department of Physiology, School of Medicine, National and Kapodistrian University of Athens, Athens, Greece; ^4^Department of Radiology, General Hospital of Athens, “G. Gennimatas”, Athens, Greece; ^5^Joint Academic Rheumatology Program, National and Kapodistrian University of Athens School of Medicine, Athens, Greece

**Keywords:** lupus, subclinical atherosclerosis, depression, anxiety, personality, sleep, fatigue

## Abstract

**Objectives:** Patients with systemic lupus erythematosus (SLE) are characterized by increased cardiovascular disease (CVD) risk as well as heightened rates of psychological distress. Since a link between psychological issues and CV morbidity has been previously suggested, the influence of psychological burden on subclinical atherosclerosis in SLE patients was investigated.

**Methods:** 71 SLE patients were assessed for the presence of subclinical atherosclerosis—defined either as carotid and/or femoral plaque formation or arterial wall thickening [Intima Media Thickness (IMT) levels > 0.90 mm by Doppler ultrasound]; personality traits, anxiety and depression, sleeping habits and fatigue levels were also evaluated by specific questionnaires including Eysenck Personality Questionnaire Scale, State-Trait Anxiety Inventory (STAI), Zung Depression Scale, Athens Insomnia Scale and Functional Assessment of Chronic Illness Therapy-Fatigue (FACIT-F). Disease related clinical and laboratory features and traditional risk factors for atherosclerosis were documented. Univariate and multivariate analysis were performed.

**Results:** SLE patients with arterial wall thickening displayed higher STAI anxiety scores (either as a current state or as a personality trait) compared to those without (49.8 ± 5.6 vs. 46.9 ± 5.4, *p*-value: 0.03 and 49.2 ± 4.4 vs. 45.7 ± 6.8, *p-*value: 0.009, respectively). In a multivariate model, trait anxiety and extraversion personality scores were found to be independently associated with arterial wall thickening and plaque formation, respectively [OR95%(CI):1.2(1.0–1.5) and 0.7(0.6–1.0), respectively], following adjustment for potential confounders. No other associations were detected.

**Conclusions:** Anxiety and extraversion personality traits have been independently associated with subclinical atherosclerosis in lupus patients, implying psychoneuroimmunological interactions as contributors in SLE related atherosclerosis.

## Introduction

Cardiovascular disease (CVD) is a well-established comorbidity of systemic lupus erythematosus (SLE), severely affecting both prognosis and mortality. Traditional CVD risk factors are present or overrepresented in SLE patients. However, the high rates of ischemic events observed are not entirely explained by Framingham scores, implemented to calculate the 10-year CV risk of an individual based on traditional CV risk factors (1). These data indicate intrinsic, disease related factors as independent contributors to SLE related atherosclerosis (2).

Depression and anxiety have been long recognized as risk factors to CVD in the general population. Chronic activation of the hypothalamic-pituitary axis, imbalance between sympathetic and parasympathetic effects on cardiac function and promotion of a pro- inflammatory milieu with higher C-reactive protein (CRP) levels as a result of imbalance between proinflammatory (IL-6, TNF-α, IFNγ) and anti-inflammatory cytokines (IL-10) have been all proposed to underlie this link (3–6). Moreover, hyperhomocystenemia (7, 8), as well as an imbalance between procoagulant molecules such as fibrinogen or factor VII and impaired fibrinolytic capacity -long implicated in atherogenetic processes- (9) have been shown to occur in the setting of chronic psychosocial stressors (10). Whether chronic autonomic stimulation results from excess in cigarette smoking, alcohol consumption and lower physical activity remains a possibility (11).

In addition, exposure to major life stressing events during childhood has been also linked to CVD, mortality and autoimmune diseases in adult life (12). Other psychological parameters that have been associated to CVD include neuroticism personality traits and sleep disturbances, with both short (1–4 h/night) and excessive sleep duration (10–18 h/night) being independently associated with CVD (13–15).

Depression, anxiety, reported poor sleep quality and fatigue have been prevalent in SLE patients compared to healthy individuals, with estimated rates of depression ranging from 30 to 38%, according to the methods used as screening tools; prevalence of anxiety tends to be higher than of depression, ranging from 37 to 40% (16). Lupus *per se* appears to influence anxiety; active disease in SLE patients [higher Systemic Lupus Erythematosus Disease Activity Index (SLEDAI) scores] has been independently associated with anxiety, irrespectively of the presence of depression (16).

Despite the well documented link between CVD and mental health issues, data exploring the impact of psychological burden in the pronounced atherosclerotic risk in the setting of SLE are relatively scarce, with only depression having been associated with heightened risk for carotid artery plaque and/or coronary artery calcification and increased carotid intima media thickness (IMT) scores (17–19).

Thus, in view of the associations observed between psychological stressors and atherogenesis in general populations, we aimed to explore whether psychopathological issues, personality traits and sleep habits are associated with subclinical atherosclerosis in a cohort of SLE patients.

## Patients and methods

### Study subjects

Seventy-one consecutive SLE patients according to the American College of Rheumatology Classification Criteria were enrolled in the study. All patients were followed at the Department of Rheumatology, General Hospital of Athens “G.Gennimatas” (Drs Mavragani CP, Giannelou M). Exclusion criteria were pregnancy, age <18 years old and renal dysfunction (serum creatinine levels>3 mg/dl, creatinine clearance <30 ml/min). The study has been approved by the Ethics Committee of the General Hospital of Athens “G.Gennimatas.” All patients provided informed consent prior to their entry to the study. The Institutional Review Board waived the requirement for written, informed consent.

All patients underwent evaluation for subclinical atherosclerosis [ultrasound measurement of intima-media thickness scores (IMT) and detection of carotid and/or femoral (C/F) plaque] at the General Hospital of Athens “G.Gennimatas.” Arterial wall thickening was defined as IMT score>0.90 mm. Demographic data, clinical features, therapeutic regimens and classical risk factors for atherosclerosis were recorded in detail, as previously described (20).

### Psychometric scales

Psychological features were assessed using self-administered psychometric questionnaires: (i) Zung Depression Scale, a validated tool for assessing depressive illness (20 items, α = 0.90) (21); (ii) State-Trait Anxiety Inventory (STAI), a questionnaire used to assess anxiety either as a personality feature (20 items, α = 0.90) or as a current state (20 items, α = 0.92) (22); (iii) Eysenck Personality Questionnaire (EPQ) Scale (86 items), a questionnaire that estimates temperamental aspects of behavior based on the three independent axes of neuroticism (α = 0.88), psychoticism (α = 0.67) and extraversion (α = 0.83) (23) (iv) Athens Insomnia Scale (AIS), a questionnaire that assesses sleep disturbances (7 items, α = 0.71) (24) and (v) the Functional Assessment of Chronic Illness Therapy—Fatigue (FACIT-F) scale in order to assess fatigue (items 13, α = 0.94) (25). All questionnaires used have been validated for the Greek population and previously implemented for psychiatric screening in autoimmune populations (26, 27).

### Statistical analysis

Two-sided Fisher's exact/chi-square and Mann-Whitney tests were implemented to compare qualitative and quantitative characteristics, respectively, between patients with and without arterial wall thickening or plaque. Correlations between IMT and anxiety scores were detected by Spearman's rho test. Univariate analyses were performed to test whether the presence of plaque formation or arterial wall thickening [high IMT (>0.9 mm)] was associated with traditional risk factors for CVD, as well as disease-related and psychological/personality features. Multivariate models including variables found to be significant in univariate analysis were constructed to detect independent associations between subclinical atherosclerosis (plaque or arterial wall thickening) and psychometric variables. A *p* < 0.05 for univariate analyses and of < 0.1 for multivariate analyses, respectively, were considered statistically significant. Data were stored in the SPSS statistical package.

## Results

### Associations between plaque formation and arterial wall thickening with traditional risk factors for CVD and disease related features

Plaque formation and arterial thickening (high IMT values >0.9 mm) were detected in 40/71 (56.3%) and 26/71 (36.6%) of SLE patients, respectively. As shown in Table 1, age, hypertension, cholesterol and triglyceride levels, as well as disease duration were found to be associated with plaque formation in univariate analysis. Increased age and body mass index (BMI), presence of hypertension as well higher levels of cholesterol, triglyceride, low density lipoprotein (LDL) and uric acid contributed to the presence of arterial wall thickening (IMT values >0.9 mm). A negative association between arterial wall thickening and current steroid dose was observed.

**Table 1:** Traditional and disease related predictors of carotid/femoral plaque and arterial wall thickening (defined as IMT scores>0.90 mm) in 71 patients with SLE included in the study.

**Classical CVD risk factors and disease related features**	**Plaque** **(*n* = 40)**	**No plaque** **(*n* = 31)**	***p*-value**	**High IMT** **(>0.9 mm)** **(*n* = 26)**	**Low IMT** **(≤ 0.9 mm)** **(*n* = 45)**	***p*-value**
Age (mean ± SD) years	52.8 ± 11.6	35.5 ± 8.2	<0.001	56.46 ± 11.0	38.76 ± 9.9	<0.001
Female sex (%)	95	93.5	ns	92.3	93.3	ns
Disease duration (mean ± SD) years	13.9 ± 9.2	6.7 ± 6.3	0.001	12.9 ± 9.5	9.4 ± 8.1	ns
BMI (mean ± SD) kg/m^2^	25.4 ± 4.2	24.0 ± 5.2	ns	26.6 ± 3.8	23.8 ± 4.9	0.004
FH of CVD (%)	20	12.9	ns	23.1	13.3%	ns
Diabetes mellitus (%)	5	6.5	ns	7.7	4.4	ns
Hypertension (%)	47.5	6.5	<0.001	61.5	11.1	<0.001
Smoking (pack/years)	14.2 ± 20.3	7.4 ± 11.9	ns	17.4 ± 23.3	7.7 ± 11.7	ns
Cholesterol levels (mean ± SD) mg/dl	191 ± 34	171 ± 34	0.027	194 ± 38	174 ± 31	0.028
Triglyceride (mean ± SD) mg/dl	131 ± 50	111 ± 62	0.047	141 ± 53	111 ± 55	0.014
LDL (mean ± SD) mg/dl	113 ± 41	100 ± 28	ns	120 ± 41	100 ± 31	0.028
HDL (mean ± SD) mg/dl	49 ± 17	48 ± 18	ns	46.9 ± 14.1	49.6 ± 19.7	ns
Uric acid (mean ± SD) mg/dl	4.9 ± 1.7	4.2 ± 1.3	ns	5.3 ± 1.6	4.2 ± 1.4	0.004
ANA ≥ 1:320 (%)	97.5	90.3	ns	96	93.3	ns
Positive anti-dsDNA titers (%)	58.3	50	ns	52	62	ns
Current steroid dose (mean ± SD) mg	9.3 ± 11.4	15.3 ± 17	ns	6.9 ± 8.0	14.8 ± 16.3	0.042
Total steroid dose (mean ± SD) gr	33.3 ± 31.8	17.1 ± 17.2	ns	26.9 ± 30.2	25.7 ± 26.1	ns
Abnormal renal function (%)	10	6.5	ns	15.4	4.4	ns
SLEDAI (mean ± SD)	9.1 ± 6.8	8.9 ± 8.8	ns	9.7 ± 7.5	8.7 ± 7.9	ns

*IMT, intima-media thickness; BMI, body mass index; CVD, cardiovascular disease; FH, family history; LDL, low density lipoproteins;HDL, high density lipoproteins; ANA, anti-nuclear antibodies; SLEDAI, Systemic Lupus Erythematosus Disease Activity Index*.

### Associations between plaque formation and arterial wall thickening with psychological/personality features

**Figure 1:**
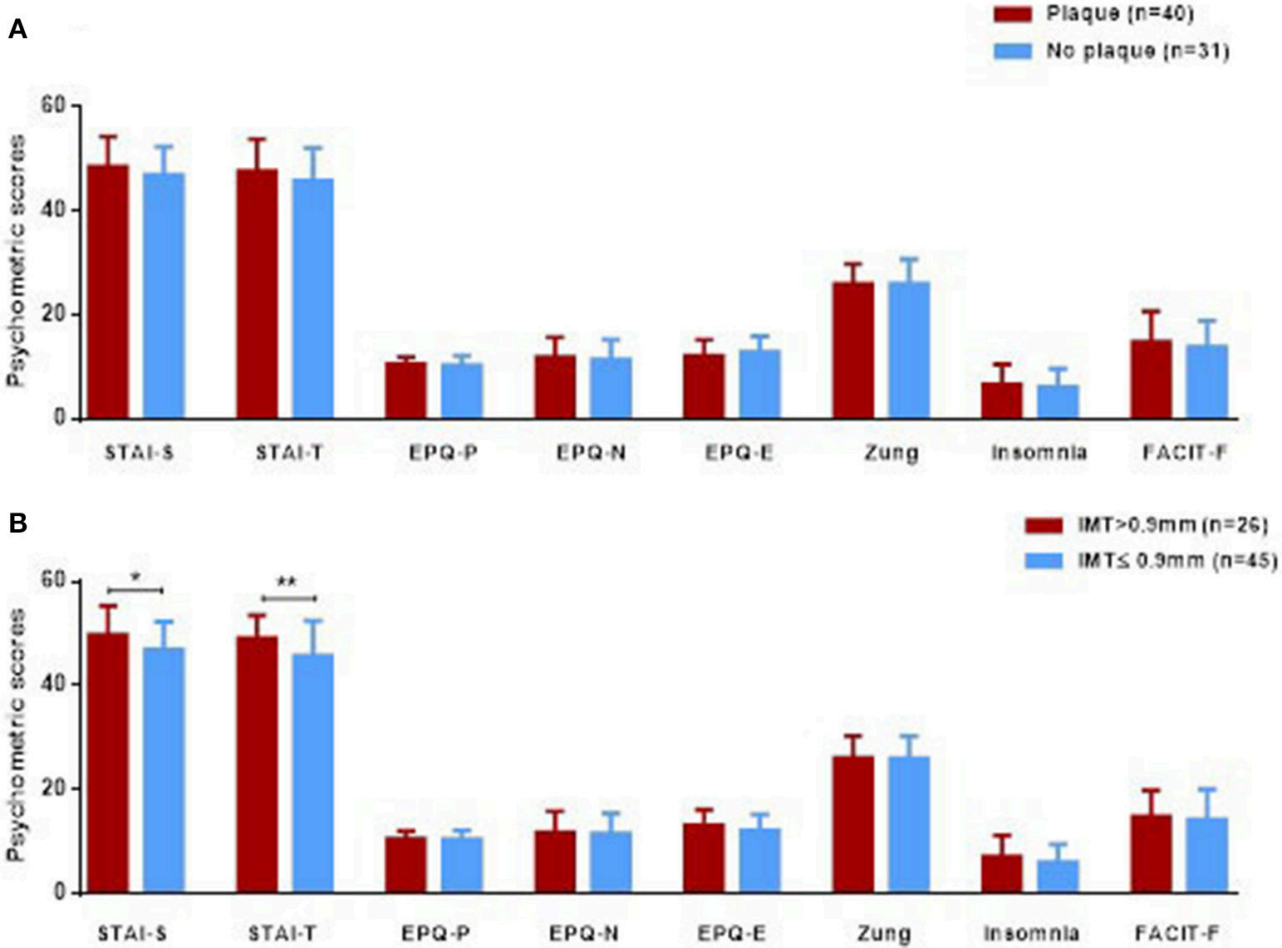
Psychometric scores (expressed in mean ± SD) in 71 SLE patients with and without plaque formation **(A)** and arterial wall thickening **(B)**, defined as IMT scores>0.90 mm).Only statistical significant differences are displayed, ^*^p < 0.05, ^**^p < 0.01. IMT, intima-media thickness; STAI, State-Trait Anxiety Inventory; EPQ, Eysenck Personality Questionnaire Scale; Zung, Zung Depression Scale; Insomnia, Athens Insomnia Scale; FACIT-F, Functional Assessment of Chronic Illness Therapy–Fatigue scale.

We next wished to evaluate whether psychological/personality features were associated with subclinical atherosclerosis in patients with SLE. While in univariate analysis, no association between plaque formation and various psychometric scores was detected (Figure 1A), multivariate modeling revealed higher EPQ-E scores (indicative of an extraverted trait) to be protective against plaque formation [OR 95% (CI): 0.7 (0.6–1.0)]; for the multivariate model, classical CVD risk factors and disease related variables emerged from univariate analysis to be associated with plaque formation, were taken in consideration (Table 1).

As shown in Figure 1B, SLE patients with arterial wall thickening (IMT>0.9 mm) displayed higher STAI-S (state) and STAI-T (trait) anxiety scores compared to those with normal IMT levels (49.8 ± 5.6 vs. 46.9 ± 5.4, *p*-value: 0.03 and 49.2 ± 4.4 vs. 45.7 ± 6.8, *p*-value: 0.009, respectively). Following adjustment for variables found to be significant in univariate analysis (Table 1), arterial wall thickening was found to be independently associated with trait anxiety scores (*OR* = 1.2, 95% CI:1.0–1.5, *p* = 0.034). No associations were detected between high IMT values and other psychometric parameters studied (depression, insomnia and fatigue).

## Discussion

The aim of the present study was to investigate whether psychological and personality features contribute to the development of subclinical atherosclerosis in the setting of lupus. To the best of our knowledge this is the first study in SLE patients to detect an independent association between markers of subclinical atherosclerosis and personality characteristics, with anxiety playing an aggravating while extraversion a protective role.

Among the psychological and personality features under investigation, only depression has been linked to subclinical atherosclerosis in lupus populations to date. Depression has been associated with a 2-fold increase in coronary artery calcification risk; notably, this association was influenced by body mass index (BMI) (18). A subsequent study from the same group detected a 4-fold higher risk for carotid artery plaque and/or coronary artery calcification in SLE patients with depression, when age, waist to hip ratio, lower education level, arterial hypertension, and CRP values were taken into account (17). In a recent report, SLE female patients with concomitant depression were shown to have increased carotid IMT- but not plaque- progression (19), while recent observations on Sjogren's syndrome implied an independent role of sleep impairment in plaque formation (27).

The lack of association with depression in the present study could be attributed to differences in the methodology implemented, as well as environmental influences. It should be noted that in all studies- including ours- the diagnosis of depression was based on single-assessment questionnaires rather than psychiatric evaluation or taking into consideration prolonged time periods.

In general population, anxiety disorders have been long related to CVD development and unfavorable CVD related prognosis, being a more potent predictor for heart disease than depression. Patients with acute myocardial infarction exhibit more often type A personality behavior (competitive and self-critical personality), which was found to be linked to higher degrees of anxiety as state and as a personality trait (28).

Mechanisms of atherogenesis are poorly understood in anxiety disorders; daily stressors and prolonged worry (anxious apprehension) have been shown to influence the activity of the autonomic nervous system (increasing sympathetic and decreasing parasympathetic activity) leading to high heart rate and low heart rate variability (shorter time interval between heartbeats) (29). Furthermore, the presence of anxiety affects traditional CVD risk factors including hypertension, obesity and increased serum cholesterol, triglyceride, and LDL-C and reduced HDL-C levels (29). Moreover, a protective role of extraversion as a personality trait has been suggested, in regard to coronary heart disease (30).

The finding that personality traits such as anxiety or extraversion are associated with subclinical atherosclerosis in lupus patients is in accord with general population studies. Since these factors can be modified by cognitive-behavioral stress reducing techniques, medication or psychotherapy sessions, these interventions could provide a complementary tool in the management of lupus related CV risk.

There are several limitations to our study. The lack of a healthy control group and the relatively small number of SLE patients enrolled warrant the confirmation of our results in larger studies. Moreover, the cross sectional design of the current study does not allow the identification of direct causal relationship between psychological burden and atherogenesis in lupus patients. On the other hand, the strengths of this study derive from the concomitant analysis of a wide range of parameters related to atherosclerosis including traditional CVD risk factors and SLE related factors, together with psychological and personality characteristics of the patients enrolled using validated psychometric tools.

In conclusion, in the present report an independent link between personality traits and CV risk among SLE individuals is revealed, pointing out the importance of a holistic approach in the management of SLE related CV comorbidities.

## Author contributions

MG and DT recorded clinical, laboratory and psychometric data from all study participants and drafted the manuscript. EA performed all ultrasonographic measurements and revised the manuscript. CM conceived, designed and coordinated the study, performed the statistical analysis and revised the manuscript. All authors read and approved the final manuscript.

### Conflict of interest statement

The authors declare that the research was conducted in the absence of any commercial or financial relationships that could be construed as a potential conflict of interest.

## Abbreviations

SLE: systemic lupus erythematosus
CVD: cardiovascular disease
CV: cardiovascular
IMT: Intima Media Thickness
C/F: carotid and/or femoral
RP: C-reactive protein
IL-6: interleukin 6
TNF-α: tumor necrosis factor-alpha
SLEDAI: Systemic Lupus Erythematosus Disease Activity Index
STAI: State-Trait Anxiety Inventory
EPQ: Eysenck Personality Questionnaire
AIS: Athens Insomnia Scale
FACIT-F: Functional Assessment of Chronic Illness Therapy-Fatigue
BMI: body mass index
LDL-C: low density lipoprotein cholesterol
HDL-C: high density lipoprotein cholesterol
FH: family history
ANA: anti-nuclear antibodies.
